# Degree of Pelvic Rotation in the Coronal Plane on Postoperative Radiographs Obtained after Total Hip Arthroplasty

**DOI:** 10.3390/jcm11216353

**Published:** 2022-10-27

**Authors:** Kuei-Lin Yeh, Tai-Yin Wu, Chiou-Shann Fuh, Chu-Song Chen, Sheng-Mou Hou, Chen-Hao Chiang, Chen-Kun Liaw

**Affiliations:** 1 Department of Orthopaedics, Ditmanson Medical Foundation Chia-Yi Christian Hospital, Chiayi City 600, Taiwan; 2 Institute of Computer Science and Information Engineering, National Taiwan University, Taipei City 106, Taiwan; 3 Department of Long-Term Care and Management, Wu Feng University, Chiayi County 621303, Taiwan; 4 Department of Family Medicine, Zhongxing Branch, Taipei City Hospital, Taipei City 103, Taiwan; 5 Institute of Epidemiology and Preventive Medicine, National Taiwan University, Taipei City 100, Taiwan; 6 General Education Center, University of Taipei, Taipei City 100, Taiwan; 7 Department of Orthopaedics, Shin Kong Wu Ho-Su Memorial Hospital, Taipei City 111, Taiwan; 8 Department of Orthopedics, School of Medicine, College of Medicine, Taipei Medical University, Taipei City 110, Taiwan; 9 Department of Orthopedics, Shuang Ho Hospital, Taipei Medical University, New Taipei City 235, Taiwan; 10 Graduate Institute of Biomedical Optomechatronics, College of Biomedical Engineering, Research Center of Biomedical Device, Taipei Medical University, Taipei City 113, Taiwan; 11 TMU Biodesign Center, Taipei Medical University, Taipei City 11031, Taiwan

**Keywords:** pelvic rotation, total hip arthroplasty, Liaw’s anteversion, hip osteoarthritis

## Abstract

There are many published cup anteversion measurements for postoperative total hip arthroplasty (THA), including Liaw’s, Lewinnek’s, and Murray’s methods. However, most measurements ignore the potential pelvic rotation on radiographs except in Liaw’s method. Without considering pelvic rotation, clinicians can miscalculate cup anteversion. Therefore, we aimed to quantify the mean degree of pelvic rotation. Herein, we collected 388 radiographs of 98 postoperative THA hips of 77 patients and measured pelvic rotation by measuring h, the horizontal displacement of the sacrococcygeal junction associated with the upper pole of the symphysis pubis, and ssd, the distance between the sacrococcygeal junction and pubic symphysis. The angle θ of pelvic rotation was defined as θ = arc sin (h/ssd) × (180°/π). The mean degree of pelvic rotation was then calculated. The standard deviation of h was 7.84 mm, and the mean ssd was 158 mm. The potential pelvic rotation was 2.50°. The *p*-values from the paired *t*-test were all >0.05 when interobserver and intraobserver errors were assessed. This is the first study to quantify the potential pelvic rotation in the coronal plane on postoperative plain radiographs. The potential pelvic rotation was too large to be neglected during the measurement of cup anteversion.

## 1. Introduction

Total hip arthroplasty (THA) is currently one of the most successful orthopedic procedures [1,2]. THA can relieve pain, restore function, and improve the quality of life in patients with hip pain due to various conditions [2,3]. In addition, the number of primary and revision THA surgeries is expected to increase over the next 20 years due to the aging of the population [4,5].

Two-dimensional plain pelvic radiography is the standard imaging modality for evaluating hip pathologies and cup position after THA [6]. Plain pelvic radiography is considered a more practical and low-cost method that confers minimal radiation exposure. In addition, the standard plain pelvic radiography performed after THA provides implant position information, such as implant loosening, postoperative infection, or insert wearing. An accurate and standard radiographic assessment of cup orientation is crucial for evaluating THA outcomes.

The importance of cup anteversion measurement after THA has been well described, including assessing the range of motion, stability, and function [7]. Furthermore, several methods for measuring cup anteversion have been described, such as Fabeck’s, Lewinnek’s, and Murray’s methods [4,8,9,10]. Because pelvic radiographs are taken by different technicians at different time points, the patient’s position could not be the same every time. However, the major difference in radiographic anteversion is associated with the difference in patient positioning [11]. To date, all of the methods did not consider pelvic rotation and could not adjust for it.

The potential pelvic rotation is neglected when post-THA radiographs are sometimes considered standard pelvic anteroposterior radiographs [12]. However, pelvic rotation is crucial for the accurate measurement of hip orientation. This study aimed to evaluate the effects of pelvic rotation and quantify the mean degree of pelvic rotation.

## 2. Materials and Methods

### 2.1. Study Design

This retrospective study was conducted in a single hospital. All patients who underwent primary THA between 2017 and 2018 were included in the study. This study was approved by the Institutional Review Board of Shin Kong Wu Ho-Su Memorial Hospital, Taiwan (Approval No. 20181005R. Approval date: 8 November 2018). Ethical approval was received before data collection. Informed consent was waived owing to the retrospective design.

### 2.2. Participants

We collected pelvic radiographs from patients who underwent THA at Shin Kong Wu Ho-Su Memorial Hospital between January 2017 and December 2018. Patients whose images had unclear anatomical landmarks, such as unclear pubic symphysis, teardrop, or sacrococcygeal junction, and those without at least two pelvic radiographs were excluded. All patients took the pelvic radiographs in the standing position. 

### 2.3. Anatomical Landmarks on Radiographic Images

The radiographic pelvic axis was defined as the vector from the center of the sacrococcygeal junction to the upper pole of the midline of the pubic symphysis. The line connecting the bilateral teardrop was considered the trans-teardrop line.

### 2.4. Pelvic Rotation Equation

**Figure 1:**
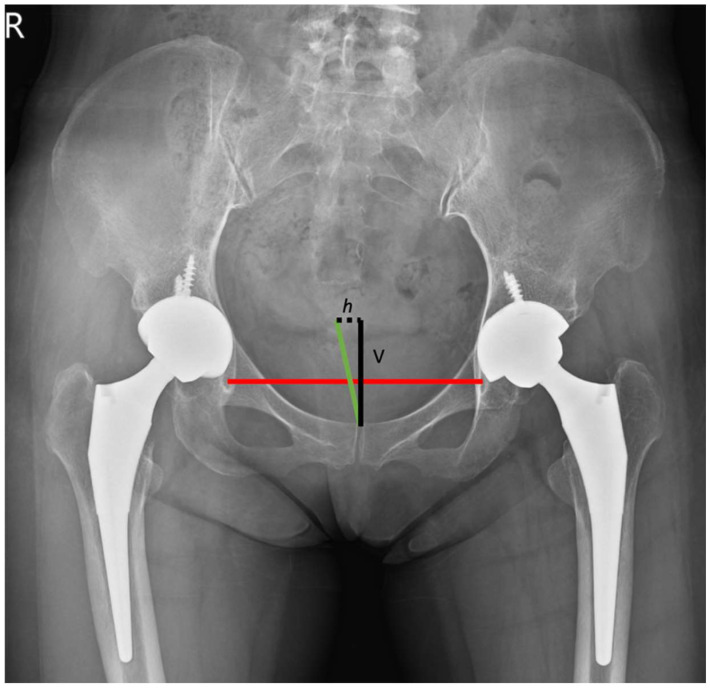
The red line represented the trans-teardrop line (TTDL), while the green line represented the distance between the sacrococcygeal junction and the pubic symphysis. The black dotted line represented h, the horizontal displacement of the sacrococcygeal junction associated with the upper pole of the pubic symphysis. The v represented the vertical displacement of sacrococcygeal junction related to the upper pole of symphysis pubis in a vertical direction.

We defined h as the horizontal displacement of the sacrococcygeal junction associated with the upper pole of the pubic symphysis. In addition, ssd was defined as the distance between the sacrococcygeal junction and the pubic symphysis. Finally, angle θ was defined as the pelvic rotation (Figure 1).

θ = arc sin (h/ssd) × (180°/π)

### 2.5. Software

**Figure 2:**
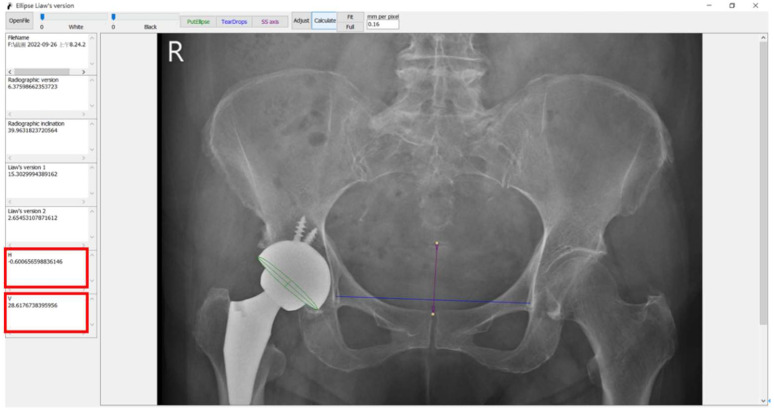
The application of the software to measure pelvic rotation. The purple line represents the radiographic pelvic axis (SCSP). The blue line represents the trans-teardrop line (TTDL). The green oval line represents the edge of the acetabular component of THA. The absolute value of H in Figure 2 was h. By applying the angle of intersection of TTDL and SCSP, we could calculate the value of ssd.

In 2019, we invented software to measure the cup anteversion using Elliversion software (Microsoft platform, Windows 11) by drawing the trans-teardrop line (TTDL), the vector from the center of the sacrococcygeal junction to the upper pole of the symphysis pubis in the midline (SCSP), and the edge of the cup [4]. In this study, we used the same software to measure pelvic rotation. By drawing two important lines, TTDL and SCSP, we could further calculate the degree of pelvic rotation (Figure 2). The equation of the pelvic rotation was applied in the software. Using this software, we could measure the h and calculate the pelvic rotation.

### 2.6. Statistical Analyses

The mean h and its standard deviation (SD) were calculated. In addition, pelvic rotation measurements for each patient were performed by two different observers. The paired *t*-test was used to analyze the interobserver and intraobserver measurement differences. Statistical analysis was performed using the SPSS version 1.0.0.1174 software (IBM Corp., Armonk, NY, USA).

## 3. Results

**Figure 3:**
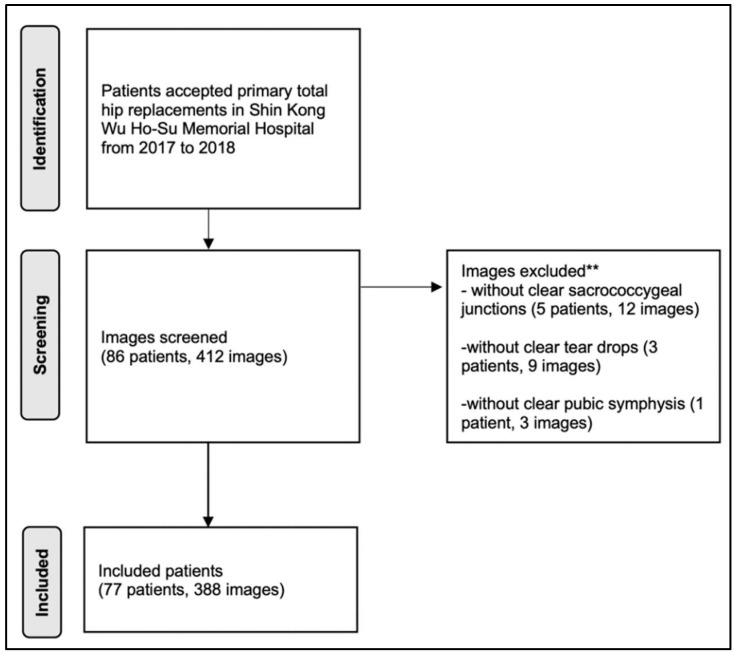
The flow chart of the patients’ inclusion. The exclusion criteria was marked “**” in the flow chart.

We collected 412 plain pelvic radiographs of 86 patients; however, we excluded 24 radiographic images due to unclear anatomic landmarks, including unclear sacrococcygeal junctions (five patients, 12 images), teardrops (three patients, nine images), or pubic symphysis (one patient, three images). Then, we analyzed 388 radiographic images from 98 postoperative THA hips of 77 patients (Figure 3). The patients comprised 50 (64.9%) women and 27 (35.1%) men, aged from 21–91 years. Of the 77 patients, 21 underwent bilateral THA. Tonnis grade II or III hip osteoarthritis was the indication for surgery in 52 patients, and 25 patients underwent surgery for Ficat stage III or IV avascular necrosis of the femoral head. All the cups of the patients were supported by screws. The surgical approaches were the anterolateral approach (Watson–Jones approach) (80 THA hips) and the posterior approach (18 THA hips). Of all 98 THA hips, 11 had cemented stems, and the remaining had non-cemented stems. Furthermore, 80 THA hips had Zimmer^®^ (Zimmer Biomet, Warsaw, IN, USA) M/L taper stems, 10 had Stryker (Stryker, Kalamazoo, MI, USA) non-cemented hip stems, and eight had Stryker cemented hip stems.

The mean (SD) of h was −0.22 (7.84) mm. The ssd ranged from 136–191 mm with a mean (SD) of 158 (12) mm. Then, we further applied 158 mm as the mean ssd to our pelvic rotation equation. The mean degree of pelvic rotation was 2.50° (ranging from 0.03° to 11.31°, with SD of 1.88°). No significant interobserver and intraobserver differences in h measurement were found, with *p*-values of 0.344 and 0.516, respectively.

## 4. Discussion

In this study, we found that potential pelvic rotation on pelvic radiographs was 2.50°. This is the first study to discuss the degree of pelvic rotation. This study revealed that standard pelvic radiographs theoretically existed; however, in real-world clinical practices, they did not exist. Therefore, we suggested the need never to ignore the potential pelvic rotation, especially when we researched its different degrees on the pelvic radiographs. 

**Figure 4:**
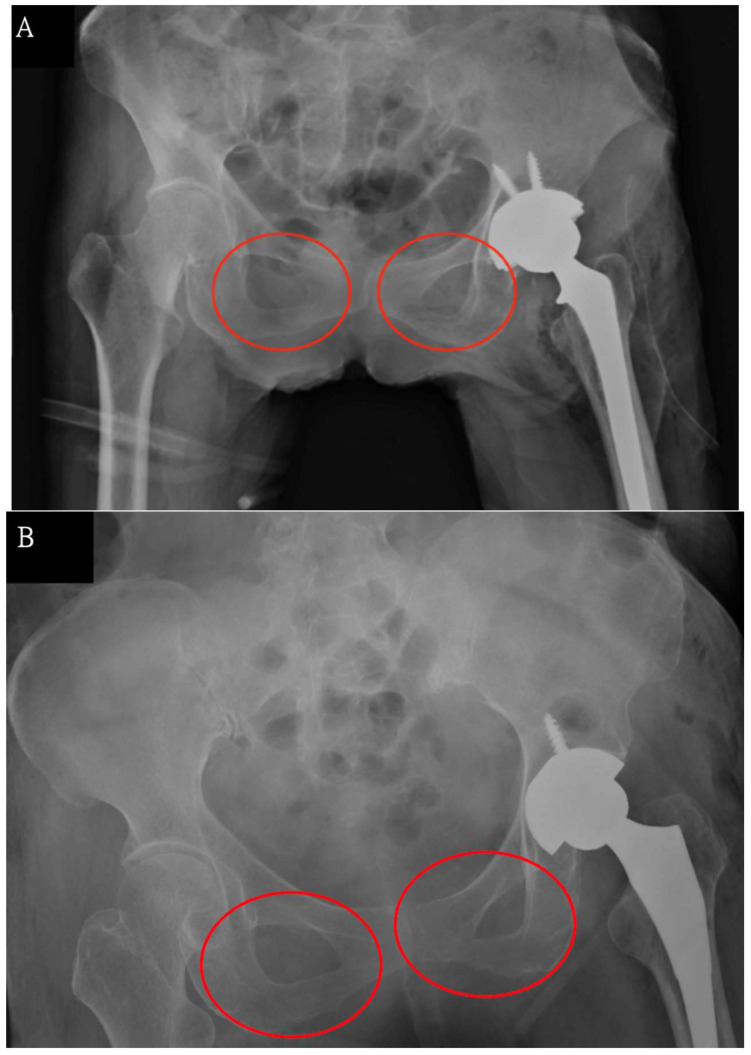
(**A**,**B**). Plain radiographs showing pelvic rotation (uneven obturator foramens, marked in red circles).

Polesello et al. have described standard pelvic radiographs previously. Standard images should be taken when the beam incident on the median line is just above the pubic symphysis with the feet rotated internally from 15° to 20° (for correction of the neck anteversion angle) so that the greater trochanter does not overlap the femoral neck [13]. However, in the clinical setting, patients sometimes do not reach the standard position for taking pelvic radiographs (Figure 4A,B). Consequently, clinicians neglect the potential pelvic rotation when taking or reading radiographs.

Previous studies have reported that orthopedic surgeons suggest that the appropriate cup anteversion ranges from 20–40° for the posterior approach and 5–25° for the anterolateral approach [14,15]. In our study, the quantified mean pelvic rotation was 2.50°. Once patients undergo THA using the anterolateral approach with the suggested anteversion of 5–25°, the potential postoperative pelvic rotation of 2.50° leads to an error of 10–50% in the anatomic anteversion measurement. Potential pelvic rotation plays an important role in cup anteversion measurement, especially in THA using the anterolateral approach.

We should never neglect the importance of anteversion measurement in clinical practice. The accurate anteversion measurement will allow us to detect small changes in acetabular version with the aid of early cup loosening detection. The early detection of tiny cup loosening may assist in realizing the unknown hip pain postoperatively. When doctors detect a small but significant cup movement they can possibly minimize the incidence of cup loosening and prosthetic hip dislocation. When early cup loosening is detected, we can warn the patient against an excessive range of motion, or take it as a signal for the need of a cup revision [4]. We would not revise a cup with an anteversion of only 2.5°, but we would pay more attention and advise the patient not to do any extreme internal rotation of hip to prevent dislocation or impingement. Thus, when a 10% to 50% error, caused by pelvic rotation exists in the anteversion measurement, the orthopedic doctors might make the wrong clinical judgement. That is why we emphasize the importance of pelvic rotation.

Furthermore, some physicians might doubt the importance of pelvic rotation. In addition, most contemporary cup anteversion measurement techniques, including Lewinnek’s, Fabeck’s, Widmer’s, and Murray’s methods, ignored pelvic rotation [6,7,10]. For example, Lewinnek’s method defined cup anteversion as arcsin (short axis of the cup/long axis of the cup) [9]. The remaining formulas for anteversion also do not consider pelvic rotation. However, there was one exception. The standardized Liaw’s anteversion measurement is the only measurement that corrects patient positioning during film acquisition [4,7]. To our knowledge, the standardized Liaw’s measurement is the most accurate method to date [4,5,16,17]. The repeated standard deviation (RSD) of standardized Liaw’s measurement by using the Ellipse method was 0.795, which was the lowest RSD among the published methods [4]. The lower the value of RSD, the more accurate the method [4]. Thus, anteversion measurements with the consideration of pelvic rotation lead to more accurate results.

Furthermore, when we defined pelvic rotation toward the opposite direction of the surgical site as positive, we found that the SD of h was 7.84. No previous study had discussed whether most pelvic rotations occur in the same direction or in the opposite direction to the surgical site. A positive h value indicates that the pelvic rotation is in the same direction as the surgical site. Conversely, a negative h value indicates that the pelvic rotation is in the opposite direction. The mean h value was −0.22 mm. This value indicates a random occurrence of pelvic rotation.

Herein, we evaluated the degree of potential pelvic rotation during postoperative pelvic radiography. Whether pelvic rotation is considered when measuring cup anteversion after THA should be clearly indicated. Furthermore, pelvic rotation affects the distance between pelvic anatomy and an x-ray film. The degree of pelvic rotation also influenced the magnification. Therefore, all the distance and degree parameters should be modified by the degree of pelvic rotation. We hope that by quantifying pelvic rotation, we can increase clinicians’ awareness of the importance of pelvic rotation. Furthermore, our results showed no significant interobserver and intraobserver differences, indicating the reliability and simplicity of our developed software.

Our study has some limitations. First, we only collected the radiographs taken when the patients were standing. The patients’ first postoperative pelvic radiographs were taken in the supine position after the patients had left the anesthesia recovery room. However, most of the subsequent radiographs were taken when the patients were ambulatory and could maintain a standing position without assistance during radiography. Our next research focus will be the effect of patient position on pelvic rotation.

Second, the different ways of projection were another limitation. Projections include perspective and orthographic projections [18,19]. Perspective projection is a linear projection where three-dimensional objects are projected on a picture plane [20]. The orthographic projection is a parallel projection in which all the projection lines are orthogonal to the projection plane [21]. The perspective projections are commonly used in clinical radiographs. However, the formula and our developed software adapt to the orthographic projection. Theoretically, the effects of the different projections might be too minimal to be considered. In clinical practice and several studies, we seldom found that researchers and doctors discriminate the difference between both projections.

There are several published studies on methods of transforming the perspective and orthographic projections into each other [22]. In our follow-up research, we will discuss the effects of the different projections when trying to get a real-world clinical pelvic rotation.

In our pelvic rotation measurement, we adapted the software we invented to increase the accuracy of measurement. Two limitations potentially existed. For one thing, the data images we collected required clear radiographic anatomy. Thus, we excluded 24 radiographic images due to unclear anatomic landmarks. For another, the software we adapted was not available online right now. In our published research, we aim to promote the use of a simple and accurate ellipse method in clinical practice, which is similar to the software used to measure pelvic rotation in this study [4]. We will provide this software online very soon for clinical use.

In conclusion, to our knowledge, this is the first study to quantify the degree of pelvic rotation in the coronal plane during postoperative THA radiography. The mean degree of postoperative pelvic rotation was 2.50°. This result shows that special attention must be paid to pelvic rotation during the measurement of cup anteversion postoperatively.

## Data Availability

Not applicable.
